# Complete genome sequence of the *Clostridium difficile* laboratory strain 630Δ*erm* reveals differences from strain 630, including translocation of the mobile element CTn*5*

**DOI:** 10.1186/s12864-015-1252-7

**Published:** 2015-01-31

**Authors:** Erika van Eijk, Seyed Yahya Anvar, Hilary P Browne, Wai Yi Leung, Jeroen Frank, Arnoud M Schmitz, Adam P Roberts, Wiep Klaas Smits

**Affiliations:** Department of Medical Microbiology, Leiden University Medical Center, Leiden, the Netherlands; Department of Human Genetics, Leiden University Medical Center, Leiden, the Netherlands; Leiden Genome Technology Center, Human and Clinical Genetics, Leiden University Medical Center, Leiden, the Netherlands; Wellcome Trust Sanger Institute, Cambridge, Hinxton, UK; Sequence Analysis Support Core, Leiden University Medical Center, Leiden, the Netherlands; Department of Microbial Diseases, UCL Eastman Dental Institute, London, UK

**Keywords:** Genome sequence, Conjugative transposon, Integrative and conjugative element, Single-molecule real-time sequencing

## Abstract

**Background:**

*Clostridium difficile* strain 630Δ*erm* is a spontaneous erythromycin sensitive derivative of the reference strain 630 obtained by serial passaging in antibiotic-free media. It is widely used as a defined and tractable *C. difficile* strain. Though largely similar to the ancestral strain, it demonstrates phenotypic differences that might be the result of underlying genetic changes. Here, we performed a *de novo* assembly based on single-molecule real-time sequencing and an analysis of major methylation patterns.

**Results:**

In addition to single nucleotide polymorphisms and various indels, we found that the mobile element CTn*5* is present in the gene encoding the methyltransferase *rumA* rather than adhesin CD1844 where it is located in the reference strain.

**Conclusions:**

Together, the genetic features identified in this study may help to explain at least part of the phenotypic differences. The annotated genome sequence of this lab strain, including the first analysis of major methylation patterns, will be a valuable resource for genetic research on *C. difficile*.

**Electronic supplementary material:**

The online version of this article (doi:10.1186/s12864-015-1252-7) contains supplementary material, which is available to authorized users.

## Background

*Clostridium difficile* is a Gram-positive, anaerobic bacterium that can asymptomatically colonize the intestine of humans and other mammals. It was originally identified as part of the intestinal microbiota of healthy infants [1]. However, when the normal flora is disturbed – for instance as a result of antibiotic treatment – *C. difficile* can overgrow and cause potentially fatal disease [2,3]. The main virulence factors are toxins A and B, that are encoded on a chromosomal region called the pathogenicity locus (PaLoc) [4], but other factors are also likely to play a role [5]. Recent years have seen an increase in the incidence and severity of *C. difficile* infections, for reasons that are only partially understood [6,7].

In 2006, the first genome sequence of a *C. difficile* strain was published [8]. This multi-resistant strain, designated 630, was isolated from a patient with severe pseudomembranous colitis and caused an outbreak of diarrheal disease in a Swiss hospital [9]. Analysis of the 630 genome sequence revealed that approximately 11% consists of mobile genetic elements [8]. The majority of these elements are conjugative transposons of the Tn*916* and Tn*1549* families called CTns, which have the ability to excise from their genomic target sites and transpose intra- or intercellularly [8,10]. Exchange of mobile elements occurs frequently and contributes to the plasticity of the genome of *C. difficile* [8,11,12]. Functions encoded on conjugative transposons can contribute to environmental adaptation and antimicrobial resistance [10,13]. In *C. difficile*, transfer of the conjugative elements CTn*1*, CTn*2*, CTn*4*, CT*5* and CTn*7* from strain 630 into a non-toxogenic strain has been shown [10]. Transfer of CTn*3* (Tn*5397*), harboring a tetracycline resistance gene, has been demonstrated between species [14,15]. CTn*1*, CTn*3*, CTn*6* and CTn*7* are related to Tn*916*, based on their conjugation module [8,13]. CTn*2*,CTn*4* and CTn*5* are all part of the Tn*1549* family, based on DNA sequence homology, and their accessory modules code for uncharacterized ABC-transporters [8,10]. Recently it has been shown that these CTn’s may also be responsible for transfer of the PaLoc on large chromosomal fragments [16].

After the demonstration of conjugative transfer from DNA from *Escherichia coli* to *C. difficile* [17], genetic tools were developed for *C. difficile*. To facilitate the genetic manipulation, an erythromycin sensitive variant was derived from strain 630 by serial passaging [18]. This strain is particularly useful for generation of insertional mutants using ClosTron that employs a retrotransposition activated erythromycin resistance marker (*erm*RAM [19]). Recently, allelic exchange methods have been developed for *C. difficile* [20,21]. The efficiency of both methods depends on the accuracy of the genome sequence for selection of target sites and recombination events. However, no comprehensive mapping of differences between the lab- and reference strains has been published to date.

The most notable phenotypic difference between 630 and 630Δ*erm,* erythromycin resistance, was found to be the result of a 2.4 kb deletion in the mobile genetic element Tn*5398* that eliminates an *ermB* gene [18]. This explains at least in part the different behavior of the two strains in a Golden Syrian hamster model of acute disease [22], as animals are generally sensitized to *C. difficile* with a clindamycin treatment (*ermB* is an rRNA adenine N-6-methyltransferase that also confers resistance to clindamycin). At a genetic level, another difference between the two strains reported to date is a duplication in the master regulator of sporulation, *spo0A*, that is apparently without phenotypic consequences [23].

In another Gram-positive bacterium, *Bacillus subtilis*, phenotypic differences between the ancestral strain NCIB3610 and widely used laboratory strains have been linked to specific genetic differences [24-26]. A detailed map of the genetic differences between the *C. difficile* strains 630 and 630Δ*erm* could therefore not only facilitate genetic manipulation, but also form the basis for the investigation of phenotypic differences between these strains.

## Results and discussion

### Reference assembly of the 630Δ*erm* genome reveals four breakpoints

**Figure 1:**
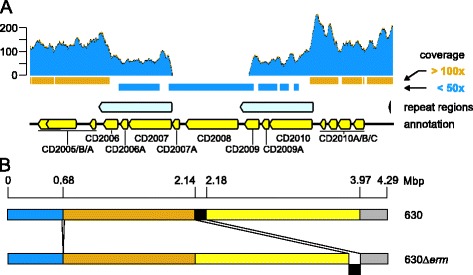
**Results of short read next generation sequencing of** ***C. difficile*** **630Δ** ***erm*** **. A**. Coverage of the region of Tn*5398* harboring the two erythromycin resistance genes (CD2007 and CD2010). Bars underneath the graph indicate a greater than 100-fold (orange) and lesser than 50-fold (blue) coverage, respectively. Reference assembly was performed using Geneious 7.1 software (Biomatters, http://www.geneious.com). **B**. Schematic representation of the breakpoint analysis (for details see Methods). Segments between breakpoints are indicated with different colors. The putative transposed element is indicated in black.

We set out to investigate differences between the laboratory strain 630Δ*erm* and reference strain 630 by performing short-read next generation sequencing on the Illumina HiSeq platform. Based on the report that the erythromycin sensitivity of strain 630Δ*erm* is due to a 2.4 kb deletion in Tn*5398*, we examined this region of the reference alignment. The analysis revealed the absence of reads mapping to the CD2007A and CD2008 genes which are located in the expected deletion [18]. Reads that mapped to CD2007 (*erm2(B)*/*ermB1*), the main erythromycin resistance determinant in strain 630 [18] are likely due to the fact that this gene shares 100% nucleotide identity with CD2010 (*erm1(B)/ermB*), which is still present. This is supported by the observation that the coverage of both these genes is approximately 2-fold lower than the immediate surrounding regions (Figure 1A). Notably, the reference assembly failed to identify the previously identified duplication in *spo0A* [23] (data not shown).

A further analysis of the reference assembly against a linearized 630 genome revealed four breakpoints (regions with discordantly mapped read-pairs). The first breakpoint is consistent with a deletion of ~70 bp. The remaining breakpoints are consistent with a transposition event, in which the transposed sequence is re-inserted elsewhere in the genome and in the inverse orientation compared to the reference (Figure 1B).

### *De novo* assembly of the 630Δ*erm* genome using third generation sequencing

**Figure 2:**
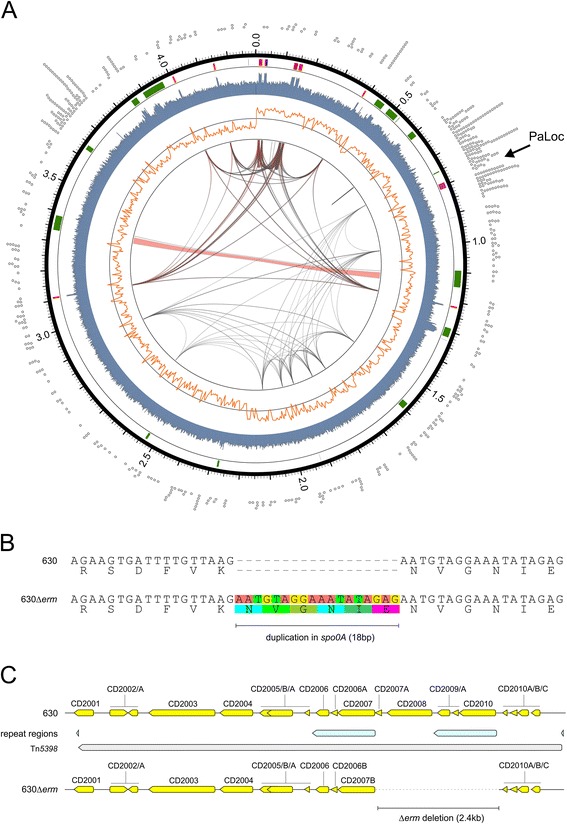
**The complete genome of** ***C. difficile*** **630Δ** ***erm*** **. A**. Overview of genomic features. Indicated are (from outside to inside); Short Tandem Repeats <500 bp (dots); rRNA (red), tRNA (blue), mobile genetic elements (green) and the PaLoc (purple); GC content per 1 kb window; GC skew (orange line) in a 5 kb sliding window; grey links represent repeats (193 repeats identified with Blast2Seq) having >95% identity and an alignment length of >500 bp; red links indicate an alignment length >2 kb. **B**. Confirmation of the 18 bp duplication in *spo0A* resulting in a 6 amino acid direct repeat [23] **C**. Confirmation of the 2.4 kb Δ*erm* deletion [18]. Open reading frames are indicated as yellow arrows, repeat elements in blue.

Based on the identification of a potential transposition event, and our previous finding that indels may have occurred that are difficult to detect using short reads, we decided to perform an unbiased, *de novo*, assembly of the 630Δ*erm* genome using single-molecule real-time sequencing. The Pacific Biosciences RSII system is capable of generating large reads, and with sufficient coverage, can generate high quality single contigs for bacterial genome sequences. We sequenced a genomic library of strain 630Δ*erm* on two SMRT cells, and validated the resulting single contig with a third SMRT cell. The resulting genome consists of 4,293,049 basepairs, with an average GC content of 29.08% and an estimated coverage of 158× (Figure 2A). We generated an annotated version of this genome by transferring the most recent version of the 630 annotation [EMBL:AM180355] [27], updating it with recent gene annotations from literature and incorporating qualifiers in the file to indicate specific features of 630Δ*erm*. The annotated sequence has been deposited under accession number EMBL:LN614756.

Satisfyingly, our unbiased approach identified the 18-bp duplication in the *spo0A* gene, encoding the master regulator of sporulation, which we previously found [23] (Figure 2B). This demonstrates that the third generation sequencing approach is superior to Illumina in identifying this type of difference. In addition, we could confirm the expected 2.4 kb deletion in Tn*5398* (Figure 2C). The sequence of Tn*5398*∆E which we determined shows 4 Single Nucleotide Polymorphisms (SNPs) compared to an *in silico* generated theoretical sequence of Tn*5398*∆E (based on Hussain *et al.*) [18]. As a result of these differences, a progressiveMAUVE [28] alignment of the Tn*5398*ΔE element from our strain with Tn*5398* of strain 630 demonstrates the deletion of CD2010 (*ermB1*/*erm(1)B*), CD2009A (ORF3), CD2009 (fragment of a putative topoisomerase), CD2008 (ORF298) and most of CD2007A. This effectively removes the region between the two copies of *ermB*. The most likely scenario by which this occurred is through recombination between the two *ermB* genes or their immediate surrounding region; the sequence information is unable to determine the exact site of recombination, as these regions are identical, and the copies of *ermB* and ORF3 in 630Δ*erm* may therefore represent hybrids of CD2007/CD2010 or CD2006A/CD2009A, respectively. To reflect the results of the alignment as well as the mechanism described above, we have chosen to rename the *ermB* gene of strain 630Δ*erm* CD2007B/*ermB* (locustag: CD630Derm_20072) and ORF3 as CD2006B (locustag: CD630Derm_20062). The resulting arrangement suggests that CD2007B is potentially expressed, as it is fused to the promoter region of CD2010/*ermB1* at the exact same location, though the strain remains erythromycin sensitive. This discrepancy has been noted since the isolation of 630Δ*erm* [18], and cannot be resolved using the sequence information from our study.

We also identified short tandem repeats (>90% nucleotide identity) up to 500 bp. Strikingly, the genome analysis revealed two regions of high repeat density (Figure 2A). The first region (approximately 0.6 Mb-0.9 Mb) includes the PaLoc that encodes toxins A and B. This region was found to be capable of transfer by a conjugation like mechanism [16] and it is tempting to speculate that the high repeat density may contribute to this phenomenon. The second region (approximately 3.6 Mb-3.75 Mb) contains many genes involved in sugar metabolism, but does not seem to be associated with annotated or characterized mobile elements. Large repeats (>95% identity and >500 bp in length) generally coincide with regions of high-GC content, and mainly reflect ribosomal gene clusters.

### Analysis of ^m6^A and ^m4^C methylation patterns of *C. difficile*

In bacteria, post-replicative addition of a methyl group to a base by a DNA methyltransferase can result in the formation of *N*6-methyladenine (^m6^A), *C*5-methylcytosine (^m5^C) and *N*4-methylcytosine (^m4^C) [29,30]. These modified bases play a role in restriction/modification systems, or may regulate cellular processes (reviewed in [30-33]).

**Figure 3:**
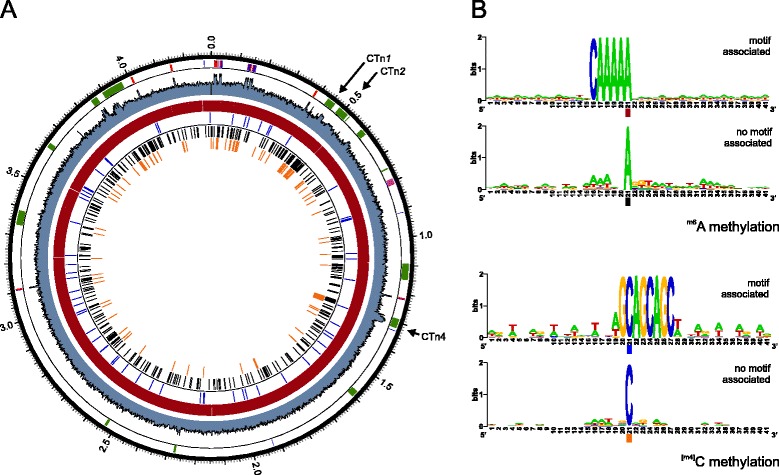
**Methylation patterns in** ***C. difficile*** **630Δ** ***erm*** **. A**. Genome wheel showing motif-associated ^m6^A methylation (red), motif associated ^m4^C methylation (blue), ^m6^A methylation not associated with a motif (black), and ^m4^C methylation events not associated with a motif (orange) in relation to GC content (per 1 kb window), rRNA (red), tRNA (blue) and mobile genetic elements (green). **B**. Sequence logos for the ^m6^A methylated sequences. **C**. Sequence logos for the ^m4^C methylated sequences.

^m6^A modifications can be identified with high confidence and the vast majority of the these modifications (7288/7687 = 95%) were associated with the motif CAAAA**A**, in which the last adenine residue is modified (Figure 3B). Previous studies identified a single methylase, M.*Cdi*25 (corresponding to CD2758) with homology to adenine specific methylases, but failed to identify its target site in restriction protection experiments [34]. We postulate that CD2758 recognizes and methylates last adenine residue the CAAAAA motif and that this is possibly the only adenine-methylase in *C. difficile* 630Δ*erm*.

There is little information on methylation of chromosomal DNA in *C. difficile*. Five methylases have been identified in *C. difficile* 630 [34], but *in vivo* methylation patterns have not been characterized. We took advantage of the pulse profiles of the Pacific Biosciences RSII reads that hold information about base modifications [35,36] to generate the first comprehensive analysis of methylation patterns in *C. difficile* (Figure 3A).

The pulse profiles of the Pacific Biosciences RSII reads also identify modified cytosines. Only a fraction of these are positively identified as ^m4^C, in part due effect of modifications that are in close proximity to each other on the pulse profiles [36,37]. We did not further investigate ^m5^C modifications, as they can only reliably be detected on the Pacific Biosciences platform after Tet1-treatment, by preparation of shorter library fragments that are not ideal for genome *de novo* assembly, and with much higher coverage than obtained in our experiment [36]. Unspecified modifications may therefore represent ^m4^C, and possibly ^m5^C or other modifications.

The SMRT Portal identified the motif G**C**AGCAGC, in which the first cytosine residue is modified, as overrepresented in the methylcytosine dataset (Figure 3B). This motif is remarkably similar to the GCWGC motif identified for the M.*Cdi*1226 methylase (CD3147) [34]. We could identify 146 instances of ^m4^C methylation and 16 of those contained the motif (11%). When a DREME search was performed [38] using 41 bp sequences centered on ^m4^C only, a highly similar motif (GCAGCR) was found in 33 instances. Moreover, none of the other motifs (see below) were specifically linked to ^m4^C modifications, suggesting that many if not all of the ^m4^C modifications are due to CD3147.

^m4^C and ^m6^A methylations that were not associated with the overrepresented motifs seemed to correspond to regions of high GC-content, including the mobile elements CTn*1*, CTn*2* and CTn*4* (Figure 3).

We also evaluated motifs previously identified as putative target sites for the other three cytosine specific methylases of *C. difficile*, M.*Cdi*633 (CD0935), M.*Cdi*587 (CD0927) and M.*Cdi*824 (CD1109) [34]. CD0935 conferred partial protection against digestion with *Bal*I (target site: TGGCCA). Our data did not show any modifications on cytosine or adenine residues of this motif anywhere in the genome (n = 396). Considering that we cannot reliably detect ^m5^C modifications in our setup, it is possible that M.*Cdi*633is an ^m5^C specific methylase. CD0927 could confer protection against *Sau*96I (target site: GGNCC) in *E. coli*, but *C. difficile* chromosomal DNA is only partially resistant to *Sau*96I digestion [34]. We found only very low levels (~0.1%) of modified cytosines for this motif (n = 3824) in 630Δ*erm*, which together with the earlier observations suggests that CD0927 is either minor ^m4^C or a ^m5^C methylase. CD1109 conferred protection against *Sma*I (which recognizes CCCGGG). We found that 6/60 (10%) of the motifs contained a modified cytosine at the third position. These modifications are likely ^m4^C’s that cannot be positively identified as ^m4^C due to adjacent modified bases.

*C. difficile* chromosomal DNA is wholly resistant to *Tse*I (target site: GCWGC) and *Sma*I (target site (CCCGGG), though we only detected modifications for ~10% of the occurrences of these motifs. This may be due to only a fraction of the methylcytosine modifications being called by the Pacific Biosciences SMRT platform in our analyses.

The function of the methylases of *C. difficile* is unknown. None seem associated with an endonuclease, indicating they are not likely to be part of a restriction-modification system. Consistent with this, no effect on conjugation efficiency was observed [34]. CD0927 and CD0935 are part of prophage 1, and CD1109 is present on the CTn*4* element, suggesting they may play a role in the biology of mobile elements.

### Comparison of the complete genome of 630Δ*erm* with strain 630 reveals SNPs, indels and rearrangements

It is likely that more than the two previously identified differences (Δ*erm* deletion and 18 bp duplication in *spo0A*) exist between strain 630 and strain 630Δ*erm*. We therefore compared our *de novo* assembled genome to the reference sequence.

We identified 71 differences between the two strains. These encompass 8 deletions (including the Δ*erm* mutation) [18], 10 insertions (including the duplication in *spo0A*) [23], 2 insertion-deletions, 50 substitutions and 1 region of complex structural variation (Additional file 1). Of these, 23 were located intergenically. This includes a 102 bp deletion which likely corresponds to the breakpoint at 0.68 Mb identified in the short read next generation sequencing (Figure 1B). A complete list of identified structural variants is available as (Additional file 1).

**Figure 4:**
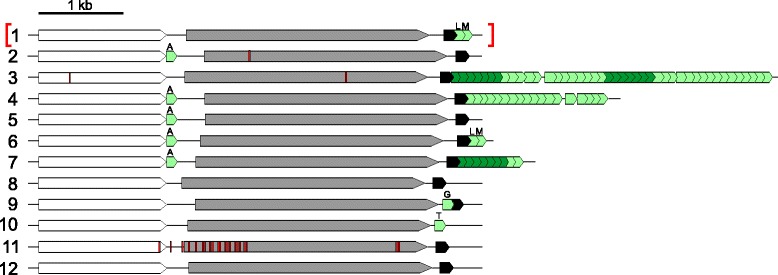
**Schematic representation of the rRNA operons and associated tRNA clusters of** ***C. difficile*** **630Δ** ***erm*** **.** Operons are numbered from 1–12 in the order they appear in the genome sequence. 16S rRNA, 23S rRNA and 5S rRNA genes are indicated with white, grey and black arrow shapes, respectively. tRNAs are indicated by green arrow shapes. SNPs between the rRNA clusters of strains 630Δ*erm* and 630 are indicated in red (for details see Additional file 1). Brackets indicate that operon 1 is unique to strain 630Δ*erm*. A cluster of tRNAs that is found multiple times associated with rRNAs (tRNA^Asn^-tRNA^Leu^-tRNA^Met^-tRNA^Glu^-tRNA^Gly^-tRNA^Val^-tRNA^Asp^) is indicated in dark green. A = tRNA^Ala^, L = tRNA^Leu^, M = tRNA^Met^, G = tRNA^Gly^, T = tRNA^Thr^. Figure is approximately to scale.

23 of the identified differences are associated with rRNA sequences. We found that strain *630Δerm* has acquired an extra ~5 kb rRNA/tRNA cluster that is inserted between CD0011 and CD0012 compared to strain 630 (Table 1, Figure 4). Copy number variations in rRNA operons have previously been noted for *C. difficile* [39] and may reflect an adaptation to favorable growth conditions in the laboratory. Similar to rRNA operon 6, this operon contains tRNA^Leu^ and tRNA^Met^ genes downstream of the 23S rRNA gene, but the intergenic spacer region (ISR) between the 16S and 23S rRNA genes does not contain a tRNA^Ala^. A detailed comparison of the ISRs of the different rRNA operons is provided as Additional file 2. A striking number of differences were found in rRNA operon 11 (Figure 4). As observed previously [40], the sequence variations cluster in the 3’ region of the 16S rRNA and 5’ of the 23S rRNA genes.

**Table 1:** **Structural variants associated with coding sequences**

**AM183055 start**	**AM183055 end**	**630Δ** ***erm*** **start**	**630Δ** ***erm*** **end**	**Type**	**Description**	**Region**	**Gene name**	**Function**	**Details**
84143		89438		Substitution	C > T	CD630_00580	*tuf1*	Elongation factor EFTu/EF1A	Synonymous
84227		89522		substitution	C > T	CD630_00580	*tuf1*	Elongation factor EFTu/EF1A	Synonymous
103225		108520		substitution	G > T	CD630_00730	*rplC*	50S ribosomal protein L3	Synonymous
610336		615631		substitution	G > A	CD630_05140	*cwpV*	Cell surface protein	Val > Ile
610480		615775		substitution	C > T	CD630_05140	*cwpV*	Cell surface protein	Synonymous
610563	610564	615859	615861	insertion	610563_610564ins	CD630_05140	*cwpV*	Cell surface protein	In frame Ala insertion
610570		615868		substitution	A > G	CD630_05140	*cwpV*	Cell surface protein	Ile > Val
610638		615936		substitution	C > T	CD630_05140	*cwpV*	Cell surface protein	Synonymous
610752		616050		substitution	G > A	CD630_05140	*cwpV*	Cell surface protein	Synonymous
610840		616138		substitution	C > T	CD630_05140	*cwpV*	Cell surface protein	Synonymous
610875		616173		substitution	C > T	CD630_05140	*cwpV*	Cell surface protein	Synonymous
755776	755800	760995	760996	deletion	755776_755800del	CD630_06320		Conserved hypothetical protein	In frame 8aa deletion in repeat region
1000995		1006274		substitution	A > G	CD630_08260		Putative ferric-uptake regulator	Thr > Ala
1391850		1397129		substitution	T > C	CD630_11900		Putative acyl-CoA N-acyltransferase	Phe > Leu
1413060	1413077	1418339	1418354	duplication	1413060_1413077dup	CD630_12140	*spo0A*	Stage 0 sporulation protein A	6aa (NVGNIE) duplication
1607458	1607459	1612756		insertion	1607458_1607459insT	CD630_13880		Putative transcriptional regulator	Restores transcriptional regulator
2044514		2049813		substitution	C > G	CD630_17670	*gapB*	Glyceraldehyde-3-phosphate dehydrogenase GAPDH	Pro > Ala
2137467	2183040	2142764	2142765	deletion	2137467_2183040del	CD630_18440		Putative adhesin	Translocation of CTn*5*, CD1844 restored
2209236		2168961		substitution	G > A	CD630_19070	*eutG*	Ethanolamine iron-dependent Alcohol dehydrogenase	Gly > Glu
2924655		2881973		substitution	C > T	CD630_25320		Aminotransferase, alanine--glyoxylate transaminase	Synonymous
3034953		2992271		substitution	C > A	CD630_26270		Conserved hypothetical protein	Gly > Cys
3080703		3038021		substitution	C > T	CD630_26670	*ptsG-BC*	PTS system, glucose-specific IIBC component	Val > Ile
3686534	3686535	3643756	3643756	insertion	3686534_3686535insA	CD630_31561		Conserved hypothetical protein	Restores conserved hypothetical protein
3967522	3967523	3924743	3970315	insertion	3967522_3967523insAM180355:g.2137467_2183040	CD630_33930	*rumA*	23S rRNA (uracil-5-)-methyltransferase	Translocation of CTn*5*; fuses *rumA* (CD3393) to CD1844A
4166495		4169292		substitution	G > A	CD630_35650		Transcriptional regulator, GntR family	Ala > Val
12347	12348	12348	17642	insertion	12347_12348ins	multiple			rRNA/tRNA cluster
2317627	2320033	2277358	2277359	deletion	2317633_2320041del	multiple			Loss of erythromycin resistance (Δ*erm*)

We focused our further analysis on the 26 variants that are associated with annotated pseudogenes or open reading frames (Table 1). A 24 bp deletion in CD0632, a conserved protein of unknown function, shortens the arginine-alanine repeat in this protein by 8 amino acids. In two cases, a single basepair insertion restores a pseudogene (CD1388 and CD3156A). This was confirmed by assembling the short read Illumina sequences against both the 630 reference genome and the *de novo* assembled 630Δ*erm* genome, as a variant was identified in the former but not the latter. CD1388 encodes a putative regulatory protein with a helix-turn-helix motif and CD3156A a conserved protein of unknown function. Interestingly, both proteins encoded by these genes were previously identified in a proteomic analysis [27], indicating that they are expressed in strain 630Δ*erm*. Two in-frame insertions were identified (an extra alanine residue in CD0514 and the published duplication in *spo0A*/CD1214). Out of 18 identified nucleotide substitutions, 9 were synonymous. These include SNPs in the gene encoding elongation factor Tu (*tuf1*/CD0058), ribosomal protein L50 (*rplC*/CD0073) and the putative aminotransferase CD2532. Strikingly, the CD0514 gene, encoding the cell wall protein *cwpV* [41,42], contains an unusually high density of mutations. In addition to the insertion and 5 synonymous mutations, it contains 2 non-synonymous but conservative mutations.

Other non-synonymous mutations are located in the putative ferric uptake regulator CD0826, the putative acyl-CoA N-acyltransferase CD1190, predicted glyceraldehyde-phosphate dehydrogenase CD1767 (*gapB*), ethanolamine utilization protein CD1907 (*eutG*), the hypothetical protein CD2627, the phosphotransferase system protein CD2667 (*ptsG-BC*) and the transcriptional regulator CD3565. In all these cases, the *de novo* assembly of the 630Δ*erm* genome was clearly supported by the short read Illumina data.

### CTn5 is present in the *rumA* gene in both 630Δ*erm* (LUMC) and 630Δ*erm*(UCL)

**Figure 5:**
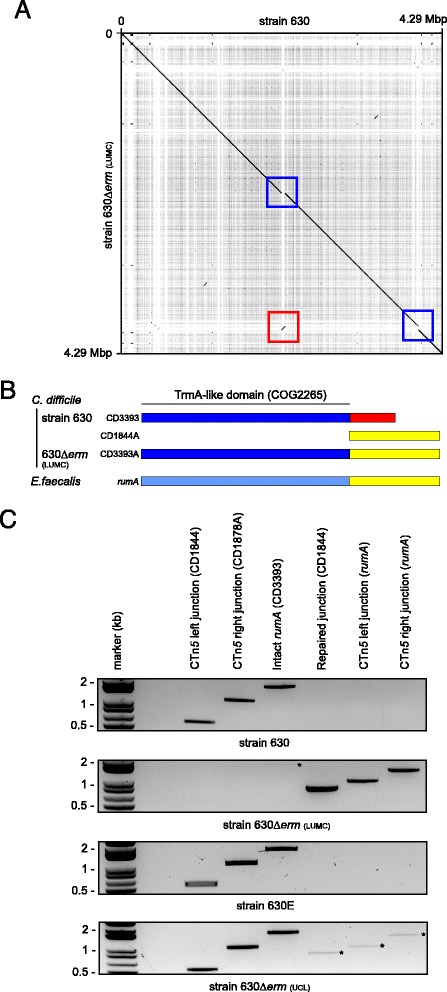
**CTn** ***5*** **is present in** ***rumA*** **in 630Δ** ***erm*** **but not 630 or 630E. A**. Dotplot of the reference sequence for *C. difficile* 630 (x-axis) versus the *de novo* assembled 630Δ*erm* sequence (y-axis), indicating the location of CD1844 and *rumA* (boxed in blue), and the CTn*5* element (boxed in red). Note the inverted orientation of the mobile element. **B**. Schematic representation of the *rumA*-CD1844A hybrid protein (CD3393A). **C**. PCR confirmation of the transposition event. For primers used see Methods and Table 2.

In an attempt to visualize the proposed transposition event (Figure 1B), we generated a dotplot of the genome sequence of our strain versus the reference (Figure 5A). It is immediately evident that the CTn*5* element seems to have excised from its original location in CD1844 (encoding a putative cell wall adhesin) and has inserted in an inverted manner in *rumA* (CD3393) in our isolate of 630Δ*erm*, for clarity hereafter referred to as 630Δ*erm*(LUMC).

To exclude that the finding represents a misassembly in the original 630 genome sequence, and confirm the presence of CTn*5* in *rumA* in 630Δ*erm *(LUMC), we performed various control PCRs (Figure 5B). In strain 630, we found CTn*5* inserted in CD1844 and confirmed an intact *rumA* gene. In contrast, in 630Δ*erm *(LUMC), we detected no product for the left and right junctions of CTn*5* in CD1844/CD1878A, indicating that the element is not present at this location. We readily amplified fragments corresponding to the left and right junction of CTn*5* when inserted in *rumA* in *C. difficile* 630Δ*erm*(LUMC), but not 630, chromosomal DNA. Interestingly, we observed a faint band corresponding to intact *rumA* even in strain 630Δ*erm (*LUMC). This indicates that a subpopulation of cells does not contain CTn*5* at this location, either because it has not inserted yet, or retains the ability to excise spontaneously as previously observed for 630 [8].

The CTn*5* insertion site identified here is located immediately downstream of CTn*7*. A similar tandem arrangement has previously been observed in two clinical PCR ribotype 001 isolates [10,43]. In another clinical isolate (RT027), which lacked a CTn*7*-like element, a CTn*5*-like element was found to be integrated at a site homologous to the target site of CTn*7* in 630 [43].

The annotation of CD3393 as *rumA* in *C. difficile* is based on homology of the predicted protein to *E. coli* RumA (also known as RlmD). This enzyme methylates a uracil nucleotide of the ribosomal RNA [44-46]. *E. coli rumA* mutants perform similarly compared with the wild type strain, in terms of cell growth, antibiotic resistance, and fidelity of translation. However, *ΔrumA* cells are outcompeted by wild type cells in growth competition assays, which may imply that ribosome function is moderately affected [46].

The translocation of CTn*5* to *rumA* has two major consequences. First, the CD1844 gene, encoding a putative adhesin is restored. Second, the *rumA* open reading frame is fused to the CD1844A open reading frame resulting into a hybrid protein (CD3393A). CD1844A shows very high similarity (e-value 1e-62, 97% identity) to the C-terminus of an *Enterococcus faecalis rumA* homolog [EMBL:EOK00135.1]. However, the homology of *C. difficile rumA* to this gene is limited to the N-terminal TrmA-like domain (COG2265) (Figure 5B). Thus, a link between these open reading frames is also found in other organisms than *C. difficile*. In order to determine what the phenotypic consequences are of the transposition of CTn*5* further experiments are required.

To further our understanding of the origin of the transposition event, we compared the location of CTn*5* by PCR in different related strains; a non-passaged isolate of the original 630Δ*erm* [18]*,* hereafter referred to as 630Δ*erm* (UCL), and another erythromycin sensitive derivative of 630, 630E/JIR8094 [47]. We found that in strain 630E the element is present in CD1844/CD1878A, identical to the reference strain, suggesting that the transposition event is not linked to the loss of erythromycin resistance. The 630Δ*erm *(UCL) strain shows prominent bands corresponding to CTn*5* at its CD1844/CD1878A location, but also a weak signal for CTn*5* at *rumA* (Figure 5C). Therefore, this isolate likely contains a subpopulation of cells with the transposition identified in this study. It is possible that CTn5 is stable at either location and the stock of the 630Δ*erm *(UCL) is non-clonal, or that CTn*5* in 630Δ*erm *(UCL) is highly mobile. During redistribution of the strain, isolates with either insertion could have been selected.

In summary, our data show that integration of CTn*5* can occur in at least two different sites in the *C. difficile* 630Δ*erm* genome, and that the element can switch between these locations during repeated passaging.

## Conclusions

The work presented here provides the first reference genome for the widely used *C. difficile* laboratory strain 630Δ*erm*, including the first analysis of major methylation patterns for any *C. difficile* strain. Our work reveals that in addition to insertion, deletions and SNPs, the CTn*5* element has moved from its original location within CD1844 to the *rumA* gene in our isolate. The observation of such a dramatic rearrangement has important implications for the redistribution of strains with highly mobile genomes and argues for complete resequencing of common lab strains in each laboratory.

## Methods

### Bacterial strains and growth conditions

Our isolate of strain 630Δ*erm* was initially obtained from the Minton lab (University of Nottingham, Nottingham, UK), that in turn received it from the Mullany lab in which it was generated. For the purpose of resequencing the strain was cultured on prereduced CLO plates (Biomerieux), after which it was ented to BHI medium (Oxoid) supplemented with 0.5% yeast extract (Fluka).

Strain 630 was originally obtained from the Mastrantonio lab (Instituto Superiore di Sanità, Rome, Italy) and its use in our lab has been described before [48]. The 630Δ*erm* strain from the Mullany lab (UCL Eastman Dental Institute, London, UK), 630Δ*erm*(UCL), was transported as a glycerol stock on dry ice. Strain 630E was a kind gift of Robert Britton (Michigan State University, East Lansing, MI, USA). All strains were cultured as described for our isolate of strain 630Δ*erm*, which is referred to as 630Δ*erm *(LUMC) where appropriate*.*

### Isolation of chromosomal DNA

For PCR analysis, chromosomal DNA was isolated using the QiaAmp Blood&Tissue kit (Qiagen) according to the manufacturer’s instructions from growth obtained after streaking out the strain directly from the glycerol stock onto CLO plates (Biomerieux). For SMRT sequencing, high molecular weight DNA was isolated from 30 mL of an overnight culture, using the Qiagen GenomicTip 500/G, according to the manufacturer’s instructions. The quality of the DNA was checked on a Nanodrop ND-200 machine (ThermoFisher), the integrity by agarose gel electrophoresis, and the DNA was quantified on a Qubit instrument (Invitrogen).

### Illumina sequencing and analysis

For Illumina sequencing, chromosomal DNA was isolated by Baseclear (Leiden, The Netherlands) from a pellet of bacterial cells derived from 50 mL culture. Data from 50 cycle 500 Mb paired-end read was delivered by Baseclear as 2 fastq files. Sequence reads have been deposited in the ENA Sequence Read Archive (EMBL:ERS550098). A preliminary analysis of the data was performed by aligning the paired-end reads to the reference genome of *C. difficile* strain 630 [GenBank:AM180355] using Geneious R7 (Biomatters, http://www.geneious.com). A more detailed analysis was performed using Stampy [49] and BWA [50]. In a routine quality control (QC) procedure on verifying the alignment, QC metrics including insert-sizes, mapped reads, unmapped reads and reads that align with a deviated pattern (DP; discordant read alignments) were examined. The case where a significant amount of reads cannot align to the reference genome indicates an undefined sequence region in strain 630Δ*erm* or a contamination of the library. In our case, a few regions with discordantly mapped read pairs (DP > 9) were identified (Additional file 3) and validated automatically (Additional file 4). Of the validated breakpoints, the first has matches with the end of the reference assembly and is therefore an artefact of assembling the reads against a linearized genome. This was confirmed by artificially breaking the circular chromosome at a different position and repeating the procedure. Visual inspection in the Integrative Genome Viewer tool [51] on the alignment track (BAM file) was used to determine the nature of the Structural Variations).

### Pacific biosciences RSII sequencing and de novo assembly

For single molecule real-time sequencing, a SMRTbell DNA template library with an insert size of ~20 kb was prepared according to the manufacturer’s specification. To this end, chromosomal DNA was fragmented with G-tubes (Covaris). Subsequently, fragmented DNA was end-repaired and ligated to hairpin adapters. SMRT sequencing was carried out on the Pacific Biosciences RSII machine according to standard protocols (Magbead loading, 1×180 min). Sequence reads have been deposited in the ENA Sequence Read Archive (EMBL:ERS550016). Sequencing reads were corrected using the HGAP pipeline [52]. Assembly was performed using Celera Assembler 8.1. We observed unbalanced coverage of two regions of approximately 18.5 kb of the reference genome. These regions were found to be nearly identical phages [16], and the unbalanced coverage therefore likely represents an artefact of the unsupervised assembly procedure using the default settings. To correct for this, the assembly was artificially broken into three contigs at these regions and was rejoined using the gap closure software PBJelly [53]. The edited assembly was then validated using reads from a third SMRT cell and polished using Quiver, a consensus algorithm that is part of the SMRT Portal. Subsequently, the consensus sequence was circularized based on the reference sequence of the ancestral 630 strain. We noted that the Pacific Biosciences consensus caller struggles with homopolymeric stretches of adenines and thymines. Therefore a correction was carried out by performing a reference assembly of the short reads from the Illumina sequencing against the reclosed genome, yielding the final genome sequence. This sequence is available from EMBL (EMBL: LN614756).

#### *In silico* analysis of the 630Δ*erm* genome sequence

To annotate the *de novo* assembled genome sequence, we first updated the most recent version of the *C. difficile* 630 genome sequence [EMBL:AM180355.1] [27] in Artemis [54,55]. Next, we imported the flat genome sequence of strain 630Δ*erm* into Geneious R7 (Biomatters, http://www.geneious.com) and transferred the annotation using the “Live Annotate and Predict” function. The annotation track was manually curated to remove duplicate or missed annotations. The resulting file was saved as a GenBank file, further polished in a text editor and Artemis and submitted to the ENA archive. Genome wheel representations were prepared using Circos [56]. Indels and single nucleotide polymorphisms were identified using the Pacific Biosciences variant caller using the genome of *C. difficile* strain 630 [8] as a reference and further validated by MUMmer 3.0 [57] and progressiveMAUVE [28]. Subsequently a list of detected structural variants was manually curated (consensus between the alignment of Illumina and PacBio reads to the reference strain and the variants identified by MUMmer and progressiveMAUVE) as concordant description of differences in complex genomic regions could not be achieved by different methods. In addition, for all large structural variants dotplots were generated using Gepard 1.30 [58] using FASTA formatted genome sequences of strains 630 and 630Δ*erm*.

To identify modified bases, kinetic signals were processed for all genomic positions after aligning sequencing reads to the final single chromosome sequence of strain 630Δ*erm*. In order to accurately identify the methylated bases, a threshold of 45 for log-transformed *P* values was used after optimizing according to its distribution and minimizing the false positive rate. Genomic positions and identity of the modifications were exported as a GFF file, and imported as a separate track in the genome sequence in Geneious R7. Subsequently, the identification of sequence motifs was performed using the SMRT Portal and sequence logos were prepared using Weblogo (http://weblogo.berkeley.edu/) [59] with 20 bp sequence flanking the modified base.

### Analysis of CTn5 translocation

Translocation of CTn*5* was confirmed by PCR using primers (Table 2) designed to amplify the left and right junctions of CTn*5* as present in the *C. difficile* strain 630, as well as the *rumA* gene (Table 1) using Q5 polymerase (New England Biolabs). Cycling conditions were: initial denaturation 98°C 30 sec, 25 cycles 98°C 10 sec/60°C 30 sec/72°C 1 min 30 sec, and a final extension 72°C for 2 mins. Products were purified (GeneJet PCR purification kit, ThermoScientific) and run on a 0.5×TAE/1.2% agarose gel with a 1 kb + ladder (Fermentas). After staining with ethidium bromide, the DNA bands were visualized on a Geldoc system (Biorad).

**Table 2:** **Oligonucleotides used in this study**

**Name**	**Sequence (5’ – 3’)**	**Description**
oWKS-1467	CGCACCAGAATGGAAAGAAG	Left junction CTn*5* ^a^
oWKS-1468	AGGCGTACACTGTGGGATAG	Left junction CTn*5* ^b^
oWKS-1469	TAGATGATGCCGTTGCTGAG	Right junction CTn*5* ^b^
oWKS-1470	AAGGTTTGGGTCTGCTGTAG	Right junction CTn*5* ^a^
oWKS-1471	CCGTTACCGTCTGTAATG	*rumA* gene^b^
oWKS-1472	AGGGCCTATAAGGTAAGC	*rumA* gene^b^

^a^The repaired junction (CTn*5* excised from CD1844) is detected with oWKS-1467 and oWKS-1470. ^b^The insertion of CTn5 into *rumA* is detected by primer combination oWKS-1468/oWKS-1472 and/or oWKS-1469/oWKS-1471.

## Additional files

Additional file 1:
**Table summarizing structural variants identified between strain 630 and strain 630**Δ***erm*** **(LUMC).**
Additional file 2:
**ClustalW alignment of the 16S-23S regions in the 630**Δ***erm*** **(LUMC) genome.**
Additional file 3:
**Table summarizing discordantly mapped read-pairs in the Illumina HiSeq reference alignment of** ***C. difficile*** **630**Δ***erm*** **(LUMC) versus 630.**
Additional file 4:
**Table summarizing validated discordantly mapped read-pairs in the Illumina HiSeq reference alignment of** ***C. difficile*** **630**Δ***erm*** **(LUMC) versus 630.**
